# Surface modification of decellularized bovine carotid arteries with human vascular cells significantly reduces their thrombogenicity

**DOI:** 10.1186/s13036-021-00277-2

**Published:** 2021-11-24

**Authors:** Eriselda Keshi, Peter Tang, Marie Weinhart, Hannah Everwien, Simon Moosburner, Nicolai Seiffert, Michael Lommel, Ulrich Kertzscher, Brigitta Globke, Anja Reutzel-Selke, Benjamin Strücker, Johann Pratschke, Igor Maximillian Sauer, Nils Haep, Karl Herbert Hillebrandt

**Affiliations:** 1 Department of Surgery, Campus Charité Mitte | Campus Virchow-Klinikum, Experimental Surgery, Charité – Universitätsmedizin Berlin, corporate member of Freie Universität Berlin, Humboldt-Universität zu Berlin, and Berlin Institute of Health, Augustenburger Platz 1, 13353 Berlin, Germany; 2 Cluster of Excellence Matters of Activity. Image Space Material funded by the Deutsche Forschungsgemeinschaft (DFG, German Research Foundation) under Germany’s Excellence Strategy – EXC 2025 – 390648296, Berlin, Germany; 3 Institute of Chemistry and Biochemistry, Freie Universität Berlin, Takustr. 3, 14195 Berlin, Germany; 4 Institute of Physical Chemistry and Electrochemistry, Leibniz Universität Hannover, Hanover, Germany; 5 Institute for Cardiovascular Computer-Assisted Medicine, Biofluid Mechanics Lab, Charité – Universitätsmedizin Berlin, corporate member of Freie Universität Berlin, Humboldt-Universität zu Berlin, and Berlin Institute of Health, Berlin, Germany; 6 Berlin Institute of Health (BIH), Berlin, Germany; 7 Department of General, Visceral and Transplant Surgery, Universitätsklinikum Münster, Münster, Germany; 8 Department of Pathology, University of Pittsburgh Medical Center, Pittsburgh, PA USA

**Keywords:** Decellularization, Recellularization, Bypass, Vascular graft

## Abstract

**Background:**

Since autologous veins are unavailable when needed in more than 20% of cases in vascular surgery, the production of personalized biological vascular grafts for implantation has become crucial. Surface modification of decellularized xenogeneic grafts with vascular cells to achieve physiological luminal coverage and eventually thromboresistance is an important prerequisite for implantation. However, ex vivo thrombogenicity testing remains a neglected area in the field of tissue engineering of vascular grafts due to a multifold of reasons.

**Methods:**

After seeding decellularized bovine carotid arteries with human endothelial progenitor cells and umbilical cord-derived mesenchymal stem cells, luminal endothelial cell coverage (LECC) was correlated with glucose and lactate levels on the cell supernatant. Then a closed loop whole blood perfusion system was designed. Recellularized grafts with a LECC > 50% and decellularized vascular grafts were perfused with human whole blood for 2 h. Hemolysis and complete blood count evaluation was performed on an hourly basis, followed by histological and immunohistochemical analysis.

**Results:**

While whole blood perfusion of decellularized grafts significantly reduced platelet counts, platelet depletion from blood resulting from binding to re-endothelialized grafts was insignificant (*p* = 0.7284). Moreover, macroscopic evaluation revealed thrombus formation only in the lumen of unseeded grafts and histological characterization revealed lack of CD41 positive platelets in recellularized grafts, thus confirming their thromboresistance.

**Conclusion:**

In the present study we were able to demonstrate the effect of surface modification of vascular grafts in their thromboresistance in an ex vivo whole blood perfusion system. To our knowledge, this is the first study to expose engineered vascular grafts to human whole blood, recirculating at high flow rates, immediately after seeding.

**Supplementary Information:**

The online version contains supplementary material available at 10.1186/s13036-021-00277-2.

## Introduction

Cardiovascular disease (CVD) affects the majority of the adult population over the age of 60, with an estimated annual mortality of 17.3 million deaths worldwide [1, 2]. It is the leading cause of peripheral vascular disease and requires percutaneous transluminal angioplasty (PTA) or bypass surgery to avoid complications such as critical limb ischemia and amputation [3]. Bypass surgery demands a biocompatible, strong, and non-thrombogenic vascular graft (VG), but autologous vascular grafts, the current gold standard, are not feasible in more than 20% of the patients [4, 5]. Prosthetic grafts have been widely implemented after *DeBakey* established the clinical usefulness of grafts made of *Dacron* (a fiber made of polyester polyethylene terephthalate) in 1950 and *ePTFE* (expanded polytetrafluorethylene) grafts two decades later [6]. However, complications such as intimal hyperplasia, calcification, thrombotic occlusion, high infection rates and biomechanical mismatch have hampered their success, especially when substituting small-diameter blood vessels [7–9].

In recent years there has been growing interest in *tissue engineering* (TE) for repurposing organs and tissues, among them vascular grafts [10]. Studies have demonstrated the crucial role of a functional luminal endothelium: it not only prevents thrombus formation within tissue engineered vascular grafts (TEVG), but also prevents intimal hyperplasia through nitric oxide released by these cells [11]. A recent meta-analysis, in which the authors found that acellular TEVG showed a lower patency rate than recellularized grafts draws attention to the importance of recellularization, surface modification, and preconditioning in achieving and maintaining superior graft patency rates [12]. Mature endothelial cells (EC) of human or xenogenous origin have been used for reconstruction of the luminal endothelial monolayer [13]. However, isolation of EC involves an invasive procedure and therefore cannot be used by default for TEVG construction [13]. Endothelial progenitor cells (ECPC) show qualities of EC upon exposure to physiological shear stress and can be isolated from the peripheral blood through routine venipuncture [14]. Moreover, ECPC isolated from peripheral blood of patients with multiple comorbidities can be used to engineer autologous VG with decreased need for immunosuppression and show a more optimal seeding efficiency when co-cultivated with human umbilical cord-derived mesenchymal stem cells (hMSC) [15]. Contact of blood with artificial biomaterials or with – in a pathophysiological context – exposed ECM activates thrombotic and inflammatory reactions [16]. Successful restoration of the endothelial lining significantly lowers thrombogenicity, a severe complication that can lead to graft failure and need for re-intervention [17, 18]. EC play an important role in mediating and impeding thrombosis through interaction via various mechanisms with platelets and leukocytes [17]. Despite widely acknowledged importance, ex vivo thrombogenicity testing is a challenging, and neglected area in the field of tissue engineering of vascular grafts [18–20]. Dynamic whole blood perfusion experimental setups are indispensable, since they correctly recapitulate the dynamics of thrombosis, but are associated with a high degree of complexity and experimental sophistication [18]. The optimal experimental setup must fulfill three main requirements: 1) Provision of flow to mimic physiological conditions that occur during thrombus formation, which might cause detachment of endothelial cells from the thrombogenic luminal surface and consequently modify the results. The implemented flow also affects the endpoint selection. 2) Whole blood as perfusion fluid and the optimal amount of anticoagulant substances. 3) It requires a balance between the surface area of the tissue engineered construct seeded with endothelial cells and the thrombogenic surface area of the connecting tubes, which is especially difficult to achieve in an ex vivo whole blood perfusion setup [18].

In the present study we mainly investigated the thrombogenic benefits of surface modification of decellularized bovine carotid arteries in an ex vivo closed loop whole blood perfusion system. We first humanized the luminal surface of the grafts by co-seeding them with hECPC derived from surgical patients and with human umbilical cord-derived MSC (hMSC). We compared two recellularization protocols and implemented daily measurements of glucose and lactate levels as **R2.3** indicators of cell proliferation, cell death, and luminal endothelial cell coverage (LECC). Based on previous results by *Ott* et al.*,* showing a 54% LECC of the pulmonary vasculature of a rat lung allowing successful orthotopic transplantation, we aimed for a LECC of greater than 50% [17]. Lastly, we perfused decellularized and recellularized vascular grafts with human whole blood in an ex vivo perfusion system and compared hemolysis values and complete blood count parameters at three different time points. To the best of our knowledge, this is the first study to evaluate the antithrombogenic impact of humanization of decellularized xenogeneic vascular grafts in an ex vivo dynamic whole blood perfusion (WBP) experimental setup.

## Methods

### Decellularization of bovine carotid arteries (dBCA)

The bovine carotid arteries were harvested from cows from a local abattoir. The animals were terminated and halved. At the time of harvesting, the carotid arteries on both sides of the neck were already exposed. Within a short time, the arteries were carefully and sharply dissected together with the surrounding tissue. Finally the vessels were rinsed in ice cold phosphate buffered saline (PBS, Biochrom, Berlin, Germany) to remove any blood remnants and transported back to the laboratory on ice. There, surplus tissue was removed and the arteries were further dissected into segments of 6–10 cm and stored in − 80 °C until further use. We approximately harvested a total of 100 carotid arteries, which had an internal diameter between 0.5 and 0.7 cm and a length of 6 cm. The decellularization protocol has been previously described [15]. Briefly, arteries were washed in double-distilled water at 4 °C in two cycles of 24 h each and later subjected to three cycles of 24 h each of enzymatic digestion (2 h of treatment with 0.05% Trypsin in 0.02% EDTA in PBS, Sigma-Aldrich, St. Louis, MO, USA), agitation in a detergent solution (4 h of treatment with Triton X-100 0.1%, Roth, Karlsruhe, Germany) and DNAse digestion (2 h of treatment with DNAse-I, 8 mg/mL, Roche Diagnostics, Risch, Switzerland) at 37 °C. In between cycles, samples were washed in PBS. Lastly, grafts were kept in sterile PBS supplemented with 1% penicillin/streptomycin (Biochrom GmbH, Berlin, Germany) at 4 °C until use.

### Cell isolation, expansion and culture

Approval for isolation of hECPC from peripheral blood of patients being treated in the Department of Surgery, Charité – Universitätsmedizin Berlin, Germany was given by the local ethical board (*Ethikkomission der Charité*, EA2/123/10). Informed consent from all patients enrolled in this study was present prior to venipuncture. hECPC and hMSC were used for recellularization. Isolation of hECPC was performed from a total of 8 patients (50% female), according to the protocol established in our previous study [15], briefly peripheral blood was obtained and blood samples were heparinized. Mononuclear cells were isolated using Biocoll (Biochrom) density gradient centrifugation and the suspension was plated on fibronectin-gelatin-coating and incubated at 37 °C and 5% CO2 for 4 days with EBM-2 medium. Afterwards, all non-adherent cells were washed off with PBS, a new EGM-2 medium was added, changed daily for 1 week and then every other day. The mean age of the patients was 50 ± 13.5 years. 50% of these were diagnosed with a malignant disease, 25% with an autoimmune disorder, and 25% with other diseases (Supplementary Table 1). The cells were expanded and cultured in *Endothelial Growth Medium*, composed of EBM-2 (PromoCell GmbH, Heidelberg, Germany) supplemented with 20% fetal bovine serum (FBS; Biochrom GmbH, Berlin, Germany), *SupplementMix* (PromoCell GmbH, Heidelberg, Germany), 500 U/mL penicillin and 500 μg/mL streptomycin (both Biochrom GmbH, Berlin, Germany) on 0.02% gelatin-coated plates (0.2 mg/ml Gelatin; Sigma-Aldrich, St. Louis, MO, USA) and used within passages 3 to 6. hMSC were purchased from ATCC (Human Umbilical Cord-Derived Mesenchymal Stem Cells, PCS-400-030, ATCC / LGC Standards; Wesel, Germany) and cultured in Dulbecco’s Medium (DMEM, 1 g/L glucose; Biochrom GmbH, Berlin, Germany) supplemented with 10% fetal bovine serum (FBS, Biochrom GmbH, Berlin, Germany), 500 U/ml penicillin, 500 μg/ml streptomycin (both Biochrom GmbH, Berlin, Germany) and 1% glutamine (Biochrom GmbH, Berlin, Germany) and used within passages 3 to 7. All cells were cultured at 37 °C in a humidified 5% CO_2_ incubator (Binder GmbH, Tuttlingen, Germany).

### Dynamic seeding and static culture of hECPC and hMSC

**Fig. 1:**
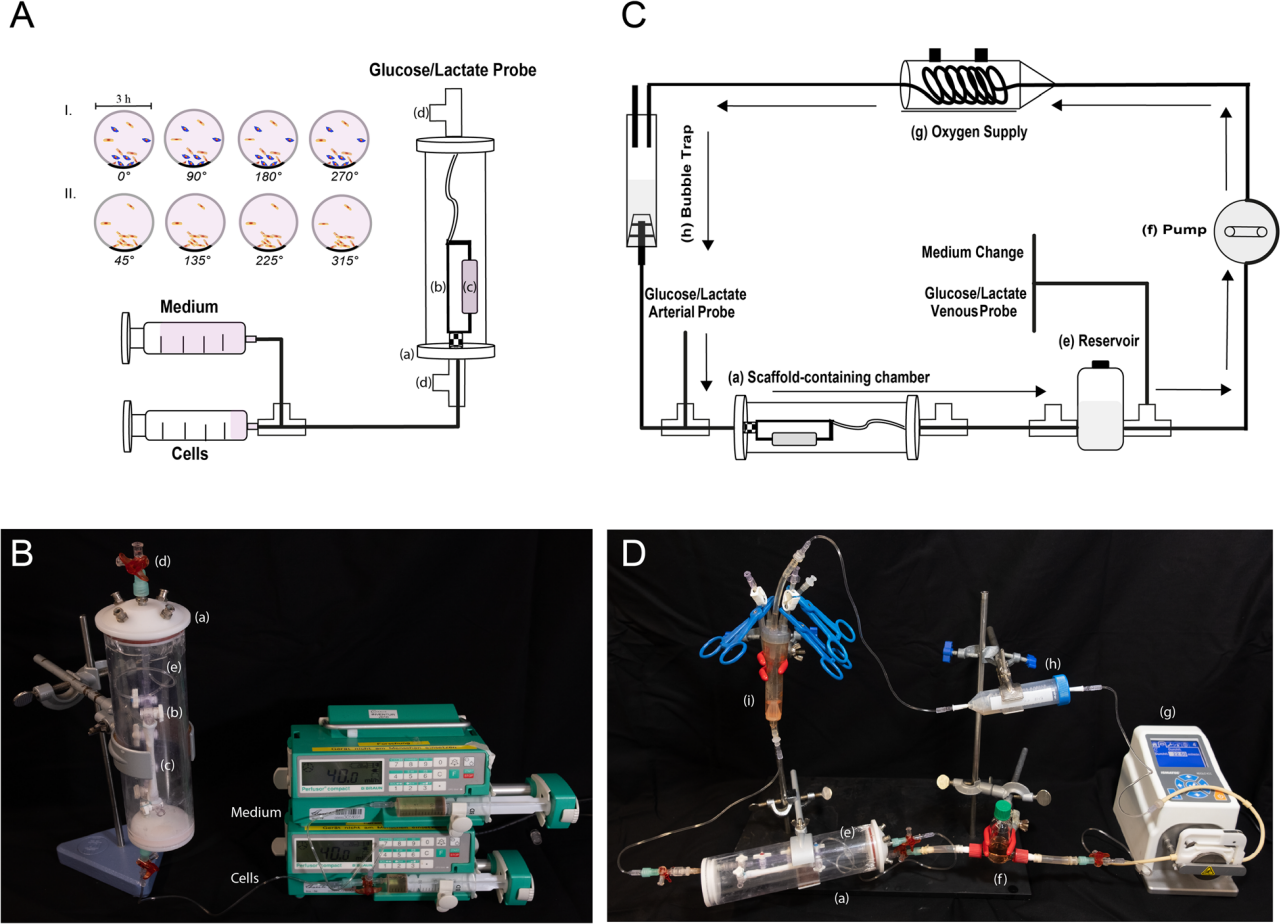
Experimental setup. For seeding, the vascular graft (c) was placed in the graft-containing portion (b) **R2.6** which is connected to the three-way stopcocks via one connecting tube (e). The bioreactor (a), was rotated vertically to expel air bubbles. Seeding was performed in two rounds: in the first four (I) both hECPC and hMSC were supplied and in the second four rounds (II) only hEPC were supplied. The bioreactor was rotated 90° and medium was drawn into a tuberculin syringe to supervise cell culture parameters after each seeding round (**A**, **B**). After static seeding, the graft-containing bioreactor chamber (a) was attached to the perfusion circuit that consists of the medium-filled reservoir (f), peristaltic pump (g), the oxygenator (h) and the bubbletrap (i) (**C**, **D**). All the inflow passes from the reservoir via the oxygenator, bubbletrap, and the tubing through the recellularized graft back into the reservoir. The arrows depict the direction of the flow

Prior to seeding, we sterilized decellularized bovine carotid arteries (dBCA) in 0.02% peracetic acid (PAA, Sigma-Aldrich, St. Louis, MO, USA) and 4% ethanol (EtOH) for 6 h at 4 °C, washed them in PBS until pH value was physiological (7.3–7.4) and soaked them in fresh EGM-2 overnight. A perfusion chamber that permits dynamic seeding, static culture, and dynamic culture was developed (Fig. 1A, B). The chamber consists of a custom-made bioreactor (a), in which the graft-containing portion (b) was placed between the two main inlets and connected to two three-way stopcocks (d) via one connecting tube. To seed dBCA, a 48-h protocol consisting of 8 × 3-h cell-seeding rounds and overnight static incubation was established. First, the components of the graft-containing chamber were assembled in a sterile culture hood. The graft (c) was embedded in the graft-containing portion of the chamber, filled with culture medium, and put in a 37 °C incubator (Binder GmbH, Tuttlingen, Germany). In the first four rounds, hECPC and hMSC were pooled and injected simultaneously using a syringe pump at a flow rate of 40 ml/h. Thereafter, cells were washed in PBS, trypsinized for 5 min, centrifuged at 300 x g for 5 min, and resuspended in the respective media. In total a density of (22.51 ± 19.75) × 10^6^ ECPC and (12.07 ± 9.86) × 10^6^ MSC was supplied. Finally the cells were pooled and collected in a 2:1 ratio in a 20 ml syringe. EGM-2 and DMEM 1:1 mixture was used for flushing the connecting loops after cell seeding. Cell seeding was carefully performed in a laminar air flow hood using a syringe pump at a rate of 40 ml/h to avoid detachment of the previously seeded cells. The graft-containing chamber was transferred to the 37 °C incubator (Binder GmbH, Tuttlingen, Germany), and cells were allowed to attach horizontally for 3 h. The chamber was connected to the oxygenation tubing (95% oxygen, 5% carbon-dioxide; DASGIP MX4/4, Eppendorf Vertrieb Deutschland GmbH, Wesseling-Berzdorf, Germany) and was rotated by 45° (longitudinal axis) each round. In the last four rounds, only hECPC were used, and the seeding was performed similarly. After each round, 0.5 ml culture medium was collected from the bioreactor for blood gas analysis (BGA) and parameters were evaluated with the standard ABL 800 analyzer (Radiometer, Copenhagen, Denmark) to monitor cell viability/proliferation.

### Flow dynamics and shear stress measurement

We calculated the shear stress the graft wall was subjected to at different flow rates during the dynamic cultivation of 10 days and during the whole blood perfusion. The investigations of the flow field were performed using the commercial CFD software STAR-CCM+ (CD-Adapco, Melville, New York), which uses a finite volume approach. The computational domain was discretized using polyhedrons in the main flow section with a base size of 0.2 mm and prismatic elements forming 12 inflatable layers on the walls for a high resolution of the wall gradients. The power quality criteria were kept within the range recommended by the STAR-CCM+ guidelines. A vessel with a diameter of 6 mm and a length of 60 mm was simulated. To ensure that the flow was fully developed, an inflow area was added in front of the vessel. The resulting mesh consists of 691,482 cells. The gradients in the flow field were discretized using the Hybrid Gauss LSQ method. A viscosity of 0.6913 mPa*s was assumed for the nutrient solution according to *Huber* et al. [21]. The density of blood was assumed to be 1.058 g/cm^3^ and for the shear dependent viscosity the Carreau-Yasuda model with the parameters described by *Abraham* et al. [22] was used. The Reynolds number was estimated at 61 for the nutrient solution and 3.5 for whole blood. Due to the low Reynolds numbers, a laminar, steady flow with constant density was assumed. Mesh independence was achieved by refining the mesh until the change in the target value of the wall shear stress was below 1%.

### Dynamic culture of Recellularized grafts

After the first 24 h of static culture, the graft-containing chamber was connected to the perfusion system. The reservoir bottle was filled with 50 ml culture medium (EGM-2: DMEM in a 1:1 ratio) and the system was flushed to eliminate air before the graft-containing chamber was embedded and the bioreactor was transferred to the incubator (Fig 1C, D). Cells were then allowed to rest quasi-statically on the luminal surface of the dBCA for 24 h before encountering pertinent flow. During this period, the culture medium was pumped at a slow flow rate of 0.3 ml/min. The next day, perfusion started at an initial flow rate of 0.6 ml/min, which was gradually elevated to 6 ml/min and 8 ml/min over 10 (*n* = 8) and 14 (*n* = 5) days. We aimed to subject hECPC to gradually increasing shear stress, while simultaneously maintaining optimal medium flow to supply all cells with nutrients. Daily replacement of half of the culture medium, control of cell viability/proliferation parameters, and graft rotation of 45° ensued. In total, 13 recellularization experiments were performed. Of those, five were dynamically cultivated over 14 days. However, the first set of analysis of cell culture parameters and endothelial coverage revealed superiority of 10-day perfusion over 14-day perfusion. Hence, 10 days dynamic culture was applied in the rest of the experiments (*n* = 8). One representative experiment was terminated after the 24 h dynamic seeding (*n* = 1).

### Histological and Immunohistochemical analysis

First, histological and immunohistochemical characterization of the dBCA was performed: The decellularized grafts were fixed in paraformaldehyde (Herbeta Arzneimittel, Berlin, Germany) for 24 h, dehydrated with an upcoming series of alcohol, and finally embedded in paraffin (Sigma-Aldrich / Merck, Darmstadt, Germany). The paraffin blocks were then sectioned at 5 μm thickness and stained with hematoxylin and eosin (H&E, Applichem, Darmstadt, Germany). Moreover, to confirm the preserved microanatomy of the dBCA, staining was performed with Rabbit polyclonal Anti-Laminin antibody diluted 1:50 (Abcam, Berlin, Germany, cat.no. ab11575), Rabbit polyclonal Anti-Collagen I antibody (Abcam, cat.no. ab6308) diluted 1:400 and Rabbit polyclonal Anti-Collagen IV antibody (Abcam, cat.no. ab6586) in 1:400 dilution. For immunohistochemical staining, 0.01 M citrate buffer was used for antigen retrieval (Agilent Technologies, Waldbronn, Germany) and 3% goat serum for blocking (Agilent Technologies, Waldbronn, Germany).

The recellularized grafts were processed as mentioned above and the paraffin blocks were then sectioned at 5 μm thickness and stained with hematoxylin and eosin (H&E, Applichem, Darmstadt, Germany). ECPC were stained with Rabbit monoclonal Anti-CD34 (Abcam, cat.no. ab110643) diluted 1:100, Rabbit Anti-CD31 antibody (Bioss Antibodies Inc., Woburn, USA, cat. no. bs-0195R) diluted 1:100, Rabbit Anti-eNOS (Abcam, cat.no. ab5589) diluted 1:100 and Rabbit anti-von Willebrand Factor antibody (Abcam, cat. no. ab6994) diluted 1:50. hMSCs were stained with Rabbit monoclonal CD90 antibody (Abcam, cat. no. ab52625) diluted 1:100. Secondary goat anti-rabbit IgG H&L (Alexa Fluor 594, ab150080) was diluted 1:400. Dako LSAB2 System-HRP (Agilent Technologies, Cat #K0675) and 3,3′-diaminobenzidine (Agilent Technologies) were used for visualization. Sections were mounted with Permount Medium (Aquatex, Merck, Darmstadt, Germany). Images were taken with a Zeiss Axio Observer.Z1 (Carl Zeiss AG, Oberkochen, Germany).

### Evaluation of accuracy of glucose and lactate as indicators of luminal coverage

At the end of the dynamic culture, estimated luminal endothelial cell coverage (LECC) based on the H&E staining of all vascular grafts was calculated. Paraffin sections from the left, middle and right portion of the graft were evaluated independently and the results were given in percentage (%). The mean coverage in percent of all three sections and the mean of the calculated percentage of the two separate evaluators was taken as the final LECC. In total, 13 experiments were evaluated. Seven of these were sole recellularization experiments, and six were the recellularized TEVG perfused with whole blood. Furthermore, we investigated the correlation between glucose and lactate on the day before finalization of the experiment with the calculated luminal coverage (*n* = 13). Finally, the sensitivity, specificity and Youden’s index were calculated to investigate performance of glucose as an indicator of LECC > 60%, the receiver operating characteristic (ROC) curve was plotted, and its area was determined.

### Designing and heparin coating of the whole blood perfusion system

The perfusion setup used during recellularization was adapted for use in our ex vivo whole blood perfusion experiments. In addition to lowering the tubing of the flow circuit to a minimum, heparin coating to further decrease thrombogenicity was performed according to a previously established protocol [23]. Briefly, the perfusion system was first statically incubated with the polymeric amine polyallylamine (0.25 mg/mL, Corline Systems AB, Uppsala, Sweden) dissolved in sodium borate buffer (250 mM, pH 9) for 15 min. Afterwards, a surface bound electrostatic complex was formed by incubation with a solution of a macromolecular conjugate of heparin (Corline Heparin Conjugate, 0.1 mg/mL; Corline Biomedical, Uppsala, Sweden) in sodium acetate buffer (0.1 M, 0.5 M NaCl, pH 4) for 60 min. After each step, the perfusion system was rinsed three times using Milli Q water. The same procedure was repeated one more time. The system was then incubated in sodium borate buffer (250 mM, pH 9) for 15 min to remove excess conjugate. Lastly, incubation in acetic anhydride solution (0.1% in 250 mM Na borate buffer, pH 10.5) for 10 min to block residual amino groups followed. All steps were performed at room temperature. If not immediately perfused, the whole blood perfusion system was filled with PBS and stored at 4 °C.

### Ex vivo whole blood perfusion of vascular grafts

Blood was drawn from six healthy volunteers aged between 18 and 65 years old with no known immunodeficiency, hereditary disease, or chronic organ dysfunction. Approval was given by the local ethical board (Ethikkomission der Charité, EA1/073/20), and informed consent was obtained from the donors.

First, the whole blood perfusion of the unseeded and seeded grafts was prepared: While the perfusion system was coated with heparin using the protocol mentioned above, a 20 mL syringe that would later be used to draw the blood was simultaneously filled with 20 μl heparin (100 U) and incubated at 37 °C for 1 h. Finally, the perfusion system was constructed. When the system and the unseeded TEVG were perfused, either the connecting tubes of both sides of the perfusion system were attached to each other or the decellularized bovine carotid artery was embedded into the perfusion system. If whole blood perfusion of the seeded graft immediately after seeding was performed, the recellularized TEVG was embedded into the system together with the TEVG-containing portion of the perfusion system. Fresh 20 mL whole blood was drawn from the donors into the previously heparinized plastic syringe and into one ethylenediaminetetraacetic acid (EDTA) prepared sample tube. The latter served as the pre-perfusion control (t0). After gentle shaking in the syringe, blood was dispensed to the system, and loops were flushed at a rate of 6 ml/min, the same flow rate applied during the 10-day conditioning period. The blood-containing reservoir was placed on a shaker and continuously shaken at 90 rpm. The VG-containing portion of the system was placed in a dish filled with previously warmed PBS to prevent drying out of the graft. The perfusion time was set at 2 h. Hemolysis evaluation via BGA (ABL 800 analyzer, Radiometer, Copenhagen, Denmark) to monitor cell viability/proliferation and collection of EDTA probes was performed for each donor separately at three time-points: before perfusion (t 0), 1 hour (t 1), and 2 hours (t 2) after perfusion. The collected probes were immediately sent to the laboratory (Labor Berlin, Charité-Vivantes GmbH, Berlin, Germany). To demonstrate the difference between seeded and unseeded VG, blood was drawn at three separate times from each donor and complete blood count parameters at three different time points were compared: a) WBP of connecting loops (w/o TEVG, *n* = 6) b) WBP of decellularized grafts (dTEVG, *n* = 6); c) WBP of recellularized grafts (rTEVG, *n* = 6).

### Histological and Immunohistochemical analysis of vascular grafts after WBP

After WBP the graft was shortly immersed in previously warmed Ringer solution (Fresenius Kabi AG, Bad Homburg, Germany) to remove residual blood and subsequently fixed in paraformaldehyde (Herbeta Arzneimittel, Berlin, Germany) for 24 h, dehydrated with an upcoming series of alcohol and finally embedded in paraffin (Sigma-Aldrich). The paraffin blocks were then sectioned at 5 μm thickness, stained with H&E (Applichem, Darmstadt, Germany) and LECC was calculated. Furthermore, we performed the same immunohistochemical stainings for hECPC and hMSC.

To histologically evaluate thromboresistance of seeded grafts and thrombogenicity of unseeded ones decellularized and recellularized whole blood perfused grafts were stained with Rabbit polyclonal *anti-CD41* antibody (Abcam Cat: ab63983) in a 1:400 dilution. Before incubating with the primary antibody, tissue slices were blocked with Serum-Free Protein Block (Agilent Technologies, Dako X0909) and Avidin/Biotin (Thermofischer Scientific). Goat Anti-Rabbit IgG H&L (Alexa Fluor 594, Abcam Cat: ab150080) diluted 1:400 was used as a secondary antibody. The CD41 staining revealed multiple cell nuclei attached to the luminal surface of the decellularized vascular grafts. To further investigate this finding, histological staining with H&E and immunohistological staining with Rabbit *anti-CD31* antibody (Bioss Antibodies Inc., Woburn, USA, cat. no. bs-0195R) diluted 1:100 and Mouse *anti-CD45* antibody (Clone X16/99, Leica Biosystems, Wetzlar, Germany) diluted 1:100 was performed. For staining with *anti-CD45* antibody, Pro Taqs Antibody diluent (BioCyc GmbH, Potsdam, Germany) was used. Finally, sections were mounted with *Permount Medium* (Aquatex, Merck, Darmstadt, Germany). Images were taken with a *Zeiss Axio Observer.Z1* (Carl Zeiss AG, Oberkochen, Germany).

### Bioburden analysis

We performed bioburden analysis following each experiment to confirm absence of contamination. To achieve that, 5 ml culture medium was mixed with 7 ml SOC (Super Optimal Broth with Catabolite Repression) medium and incubated for 24, 48, 72, and 96 h at 37 °C and 150 RPM. SOC/Sputum mixture was used as the positive control. Absorbance was measured at 425 and 600 nm using NanoDrop 2000 C Uv-Vis Spectrophotometer (Thermo Fisher Scientific). SOC/PBS mixture was used as a blank.

### Statistical analysis

All data were analyzed and visualized using GraphPad Prism version 7.00 (GraphPad Software, La Jolla, California USA). Gaussian Distribution was calculated using the Shapiro-Wilk normality test. Data are expressed as the mean ± standard error of the mean (SEM). Ordinary One-Way ANOVA (data with Gaussian Distribution) followed by Tukey’s Multiple Comparison Test or Kruskal-Wallis Test (data without Gaussian Distribution) followed by Dunn’s Multiple Comparison Test was performed to compare cell culture parameters. ROC analysis was performed to determine the sensitivity and specificity of glucose as an indicator of LECC and the cut-off value was determined using the Youden Index. Student’s paired t-test was performed to compare between arterial and venous BGAs. Pearson’s correlation coefficient (*r*) or nonparametric Spearman’s correlation coefficient were used to investigate the association between BGA parameters and histological evaluation. Data is presented as 95% confidence interval (CI). A *p*-value of less than 0.05 was considered significant.

## Results

1.1. *Wall Shear Stress in the Simulated Dynamic Cultivation and WBP Model*

**Fig. 2:**
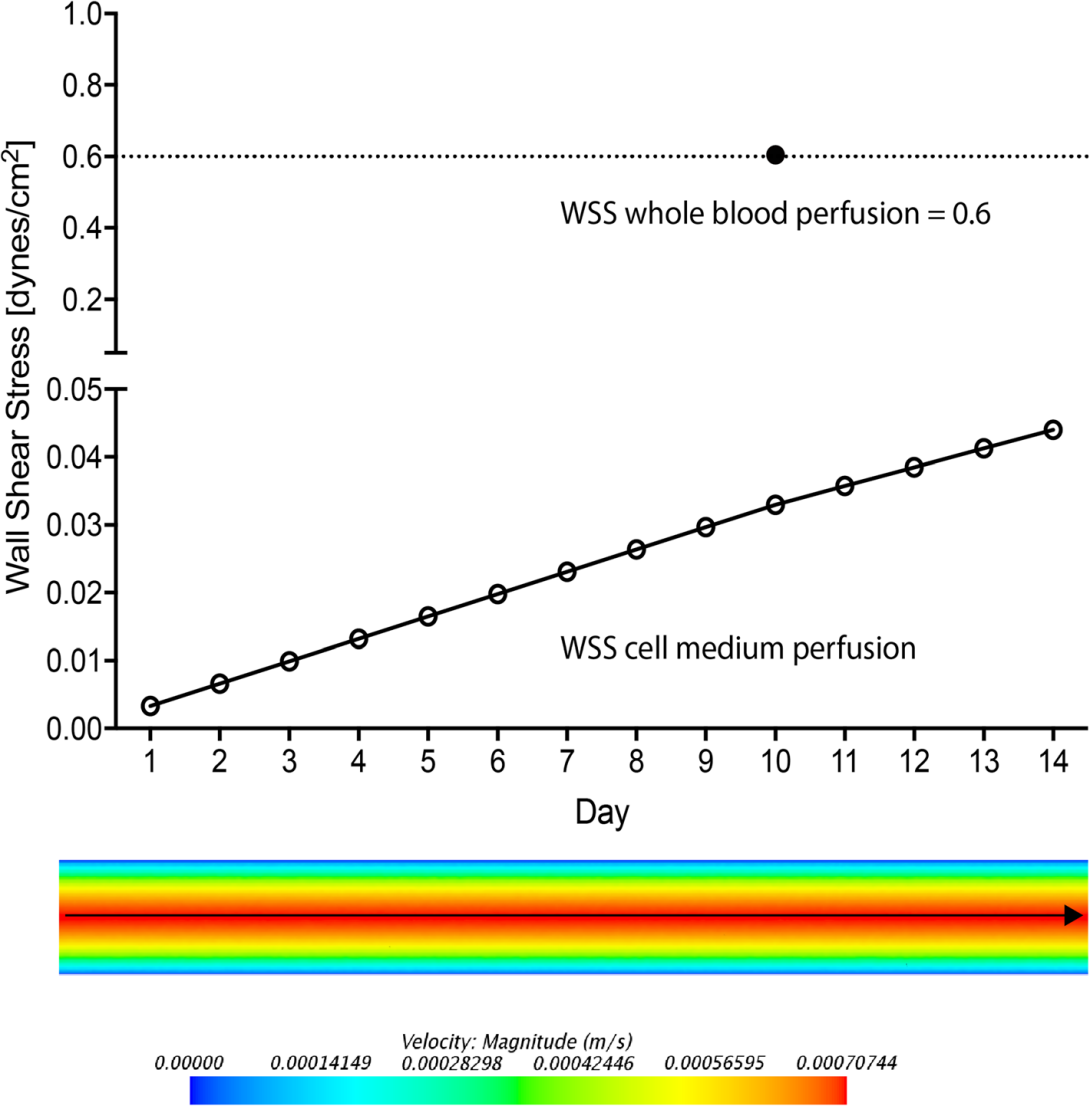
Wall shear stress (WSS) during perfusion with cell medium and whole blood. To calculate the WSS exerted on the vascular graft with each flow rate, a vessel with a diameter of 6 mm and a length of 60 mm was simulated. During dynamic cultivation with cell medium we applied sub-physiological WSS values of minimum 0.0033 dynes/cm^2^ and maximum of 0.033 dynes/cm^2^ for the 10-day culture and 0.044 dynes/cm^2^ for the 14-day culture, as depicted by the gradually increasing line. However, we adjusted the WSS to a physiological venous WSS of 0.6 dynes/cm^2^ during whole blood perfusion, as depicted by the dot

We measured the wall shear stress (WSS) exerted on the vascular graft during the dynamic cultivation using a simulation setup. Aiming to supply cells with nutrients in a continuous manner, without skipping any regions and to avoid cell detachment due to abruptly increasing flow rates we chose to supply the vascular grafts with a sub-physiological shear stress during the dynamic cultivation with cell medium. The maximal flow velocity in our simulated vascular graft was registered in the central part of the vascular lumen at a value of 0.0007 m/s (Fig. 2). We applied an initial shear stress of 0.0033 dynes/cm^2^, which we gradually elevated to 0.0330 dynes/cm^2^ during the 10-day cultivation and to 0.0440 dynes/cm^2^ during the 14-day cultivation (Fig. 2). Nonetheless, we aimed at physiological venous shear stress when perfusing with whole blood for 2 h and adjusted the wall shear stress during this time to 0.6 dynes/cm^2^ (Fig. 2). Importantly, aiming for an arterial WSS during whole blood perfusion would lead to very high hemolysis rates within a very short amount of time, as it can be seen occurred within 2 h during WBP under venous WSS.

### Confirmation of Decellularization

**Fig. 3:**
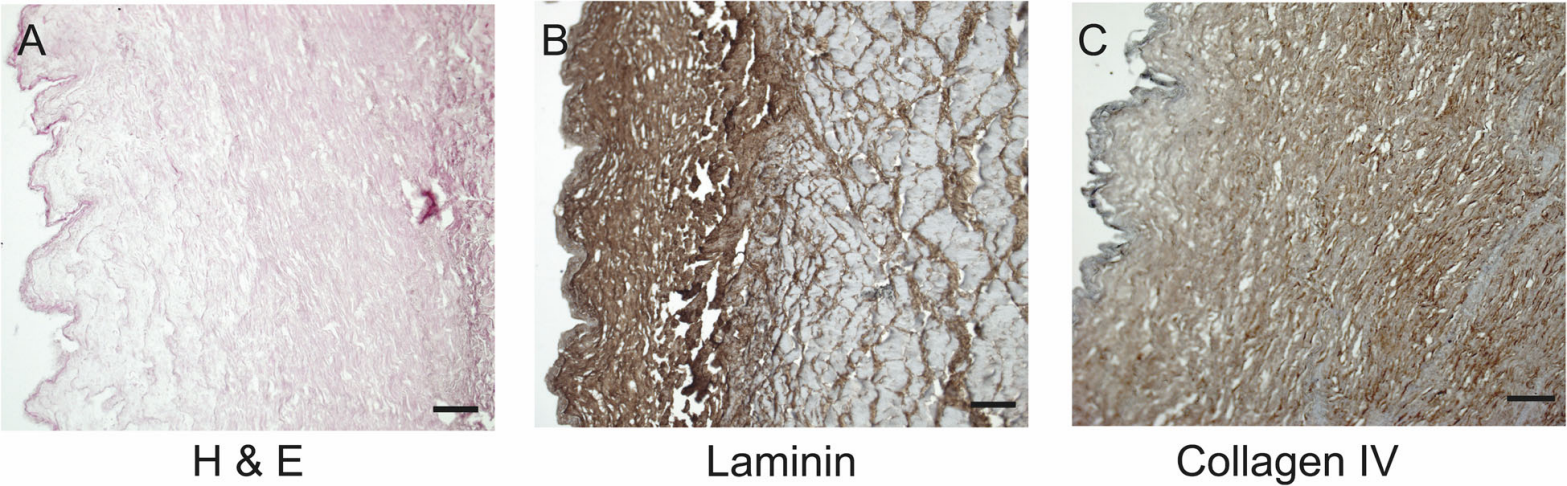
Histological characterization of decellularized bovine carotid artery grafts. Hematoxylin and Eosin (H&E) staining confirmed lack of cell nuclei in the dTEVG after decellularization with 0.05% Trypsin/ 0.02% EDTA, 0.1% Triton X-100 and DNAse (**A**). Furthermore, staining with Rabbit polyclonal Anti-Laminin antibody (**B**), Rabbit polyclonal Anti-Collagen IV antibody (**C**) revealed crucial components of the extracellular matrix (ECM) by a brown staining such as laminin and collagen type I and IV, which were preserved during decellularization. Scale bar represents 50 μm

We decellularized tubular bovine carotid arteries with an internal diameter of 0.5–0.7 cm and an approximate length of 6 cm. In the experimental work preceding the current one we performed an extensive comparison for different stainings between native and decellularized bovine carotid arteries, and thus did not repeat here [15]. Moreover, in the same work we performed an extensive biochemical and mechanical characterization of the dBCA [15]. In the current study we could show that the vascular grafts preserved their macroscopic integrity throughout the decellularization process. The H&E staining of the grafts revealed lack of residual cellular structures, thus confirming efficient decellularization (Fig. 3A). Moreover, immunohistochemical staining with Laminin and Collagen IV antibodies revealed preserved graft microanatomy (Fig. 3 B, C).

### Cell viability and proliferation during seeding and static culture

To monitor cell viability and cell culture stability BGA parameters were measured every 3 h during the 24-h seeding period (t3, t6, t9, t12, t15, t18, t21, t24). While pH, pO_2_, pCO_2,_ Na^+,^ and K^+^ were used as indicators of cell culture stability, glucose and lactate served as indicators of cell proliferation and viability.

**Fig. 4:**
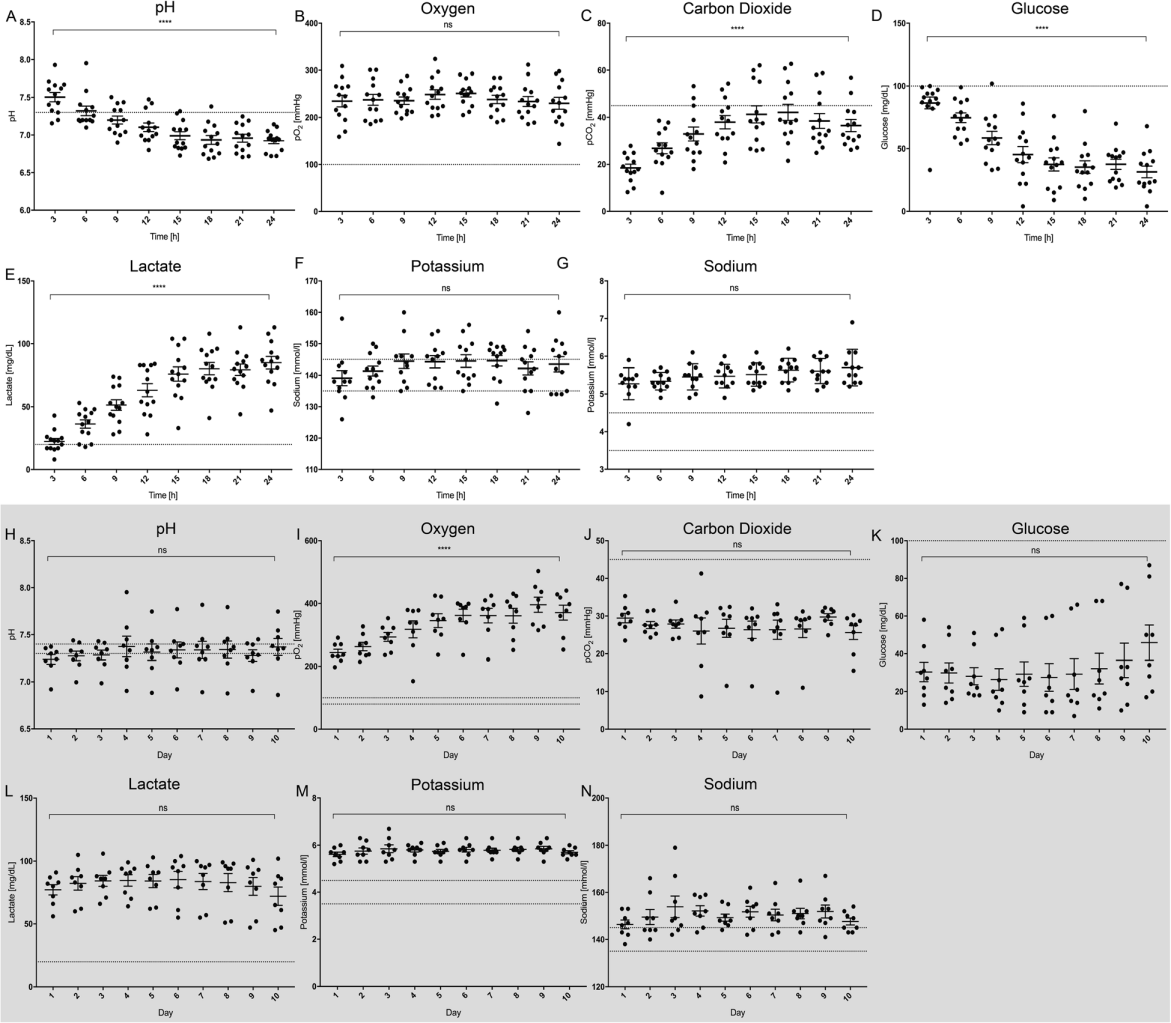
Cell culture parameters during seeding, static cultivation and dynamic culture. Parameters during static culture: Supervision of pH during static cultivation every three hours revealed the values gradually decreased (**A**). While pO2 values maintained stable (**B**), pCO2 values significantly rose (**C**). Glucose (**D**) and lactate (**E**) values respectively decremented and incremented significantly during the static cultivation, while Na^+^ (**F**) and K^+^ (**G**) concentrations preserved stability throughout its duration. (****, *p* < 0.0001). Parameters during dynamic cultivation: pH which preserved stability (**H**), pO_2_, which significantly incremented presumably due to increasing flow rates (**I**) and pCO_2_ (**J**), glucose (**K**), lactate (**L**), Na^+^ (**M**) and K^+^ (**N**) which also preserved stability throughout the whole duration of the dynamic culture. (****, *p* < 0.0001). The dots represent different experiments

The pH gradually decreased between t3 and t24 of cultivation and showed a statistically significant change at all time points (t3 vs. t24: 7.50 ± 0.22 vs. 6.92 ± 0.14, *p* < 0.0001, Fig. 4A). pO_2_ remained stable throughout all time points (t3 vs. t24: 234.85 ± 44.70 vs. 230.153 ± 45.42 mmHg, *p* = 0.8280, Fig. 4B). However, pCO_2_ behaved differently, showing significantly increasing levels (t3 vs. t24: 18.46 ± 5.87 vs. 36.51 ± 9.31 mmHg, *p* < 0.0001, Fig. 4C).

Although glucose concentration remained constant during the first three time-points (t3 vs. t9: 86.54 ± 17.04 vs. 58.62 ± 19.28 mg/dL, *p* = 0.6565), it continuously decreased after each cellular injection showing a statistically significant difference between the first and last injection, and all time-points after t9 (t3 vs. t24: 86.54 ± 17.04 vs. 31.46 ± 16.78 mg/dL, *p* < 0.0001, Fig. 4D). In contrast lactate gradually increased, showing an insignificant change only during the first 3 h of static cultivation (t3 vs. t6: 22.38 ± 8.57 vs. 36.23 ± 11.84 mg/dL, *p* = 0.3649; t3 vs. t24: 22.38 ± 8.57 vs. 85 ± 17.59 mg/dL, *p* < 0.0001, Fig. 4E). Moreover, no significant correlation could be detected between glucose and lactate, and between lactate and carbon dioxide (data not shown). These results indicate ongoing cellular activity inside the VG and were also monitored during dynamic cultivation to indicate luminal endothelial coverage. These results indicate ongoing cellular activity inside the VG and were also monitored during dynamic cultivation to indicate luminal endothelial coverage. Furthermore, both sodium (Na^+^) and potassium (K^+^) ion concentrations remained stable during the whole reseeding process (Na^+^, t3 vs t24: 139.09 ± 7.99 vs. 143.50 ± 8.32 mmol/L, *p* = 0.5005, Fig. 4F; K^+^, t3 vs. t24: 5.27 ± 0.42 vs. 5.70 ± 0.48 mmol/L, *p* = 0.0611, Fig. 4G).

### Cell viability and proliferation during dynamic culture

During dynamic perfusion, both arterial (afferent of the graft) and venous (efferent of the graft) BGA parameters were evaluated. However, since no significant differences were found between the two, we chose the values of the venous BGA for the rest of the analysis.

To find the optimal duration for dynamic cultivation 14-day (*n* = 5) and 10-day (*n* = 8) perfusion protocols were compared. Although most parameters were comparable with both protocols (Supplementary Fig. 1A), glucose and lactate gradually shifted to baseline levels towards the end of the perfusion in the 14-day protocol, strongly suggesting cell detachment (Supplementary Fig. 1B). This implication is in agreement with the inferior average LECC of these grafts, which was estimated as 33% ± 34.2% from the evaluation of stained tissue slices. Detailed analysis of the data revealed a **R2.5** minimal, statistically not significant rise of glucose levels from day 10 to day 14, a finding that marks day 10 as the turning point in cell viability inside the graft (d10 vs. d14: 70.00 ± 26.01 vs. 80.80 ± 14.81 mg/dL, *p* > 0.9999, Supplementary Fig. 1B). Based on these data, we implemented a 10-day ex vivo dynamic cultivation protocol for our further experiments (*n* = 8). Of note, the apparent lack of correlation between glucose and lactate on day 14 and the estimated LECC can be attributed to the low sample size (glucose: *r* = − 0.5, CI: − 0.9635 to 0.6523, *p* = 0.346; lactate: *r* = 0.7550, CI: − 3.82 to 0.98, *p* = 0.14, Supplementary Fig. 1C). pH mainly remained physiological and stable during the whole 10-day perfusion period (*p* > 0.999, Fig. 4H). Despite a statistically significant increase in pO_2_ when comparing levels on day 1 with all other time points, which can be explained by the increase in flow rate, pO_2_ and pCO_2_ preserved their stability Fig. 4I, J). Importantly, both glucose and lactate remained stable throughout perfusion, implying that cells did not overgrow or detach inside the graft (glucose, d1 vs. d10: 28.13 ± 12.82 mg/dL vs. 45.88 ± 26.52 mg/dL, *p* > 0.9999, Fig. 4K; lactate, d1 vs. d10: 77.13 ± 11.67 mg/dL vs. 72.00 ± 20.62 mg/dL, *p* > 0.9999, Fig. 4L). As expected, sodium and potassium ion concentrations behaved similarly (Fig. 4M, N).

### dBCA supports growth of hECPC and hMSC

We performed histological and immunohistochemical staining of the statically cultivated (rTEVG) and 10-day (dTEVG-10d) and 14-day (dTEVG-14d) dynamically cultivated TEVG .

**Fig. 5:**
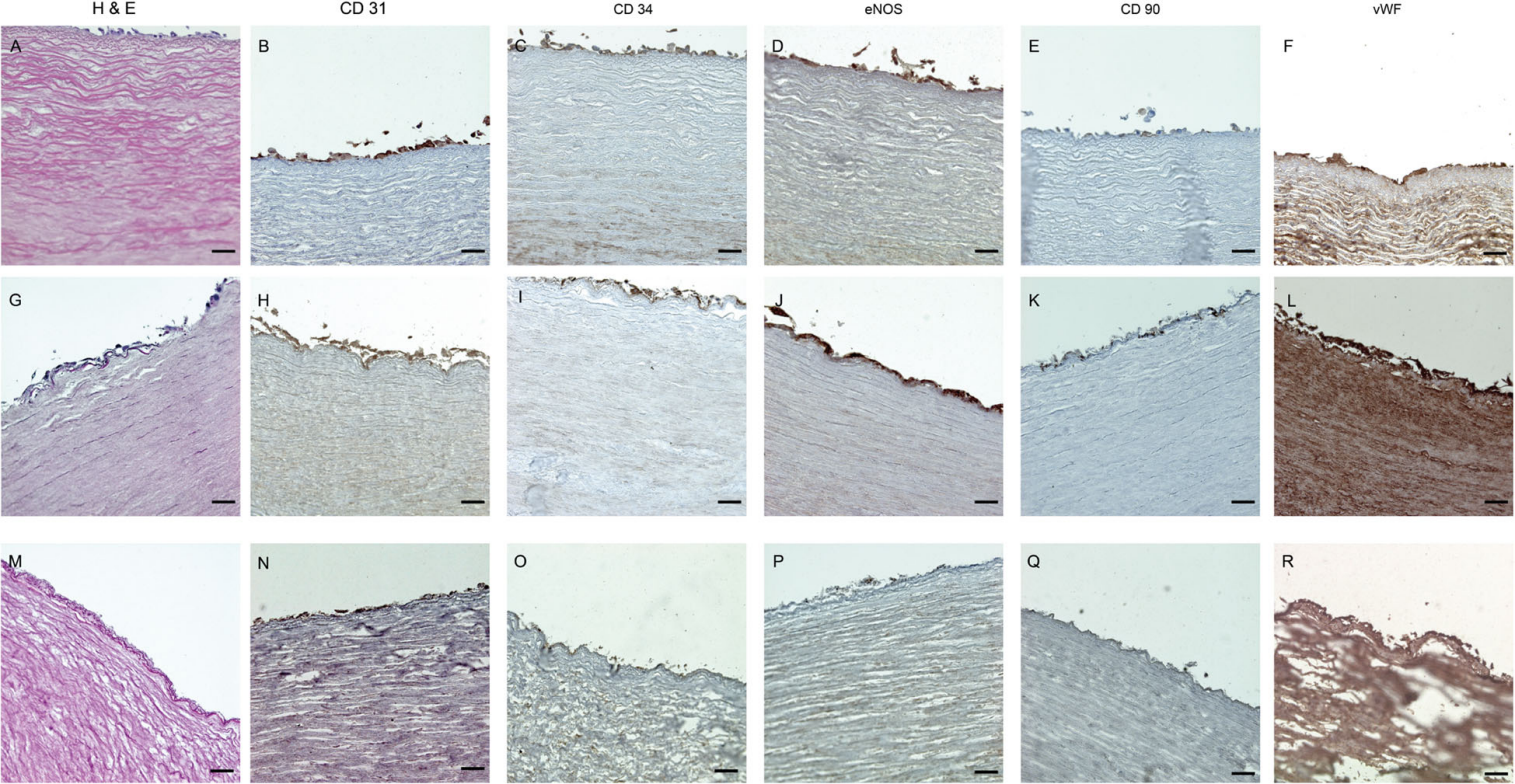
Histological and immunohistochemical evaluation of recellularized vascular grafts. Histological evaluation of the seeded, statically cultivated and immediately examined vascular graft (rTEVG-s) with H&E staining revealed multiple, morphologically round cells discontinuously attached to the basal membrane of the vascular graft (**A**). Further immunohistochemical staining with important endothelial cell markers such as CD31 (**B**), CD34 (**C**), eNOS (**D**) and vWF (**E**) revealed the majority of the cells were of endothelial phenotype. A few cells also stained positive for CD90, indicating a much lower number of hMSC dispersed within the hEPC (**F**). Histological evaluation of the dynamically cultivated vascular grafts (rTEVG-10d) displayed morphologically elongated cells due to introduction of gradually increasing medium flow and shear stress (**G**). Further characterization with CD31 (**H**), CD34 (**I**), eNOS (**J**) and vWF (**K**) revealed that while the cells stained strongly for eNOS, CD31 and vWF, staining with CD34, a marker of immature EPC was much weaker, a finding which was interpreted to point to cell maturation. Moreover, staining with CD90 antibody revealed scarce hMSC dispersed and attached to the basal membrane (**L**). Lastly, histological characterization of the vascular grafts perfused over 14 days (rTEVG-14d) revealed morphologically irregular cells (**M**), which stained slightly positive for CD31 **(N)** and vWF (**O**) and did not stain for CD34 (**P**), eNOS (**Q**) and CD90 (**R**), findings in line with the cell culture parameters, that pointed to excessive cell detachment and death after the 10th day of cultivation. Scale bar represents 50 μm

One representative experiment was finalized after 24-h seeding and static cultivation. Histological staining of this graft revealed intact, morphologically round cells mostly tightly attached to the luminal surface of the basal membrane (Fig. 5A). Immunohistochemical staining for CD31, CD34, eNOS and vWF confirmed the endothelial origin of the majority of these cells (Fig. 5B, C, D, E). Moreover, the staining revealed few CD90-positive hMSC irregularly arranged within the hECPC (Fig. 5F). As expected, lack of flow and shear stress accounted for both the round cellular morphology and for the numerous cell clumps located within the lumen of the TEVG (data not shown). On the other hand, H&E staining of the 10-day dynamically cultivated VG revealed an intima repopulated with a morphologically flat, continuous, tightly attached cellular monolayer (Fig. 5G). As staining for CD31, CD34, eNOS and vWF confirmed, the majority of the cells were of endothelial phenotype (Fig. 5H, I, J, K). Moreover, it appears the physiological shear stress accounted for a maturation of hMSC into endothelial cells, as suggested by the decline in CD90 positive cells (Fig. 5L). Finally, histological staining of the 14-day dynamically cultivated VG, revealed flatter cell morphology than the 10-day perfused VG. As predicted, this cellular monolayer appeared to be rather discontinuous, confirming the cell detachment earlier implied by the BGA parameters (Fig. 5M, N, O, P, Q). Importantly, significantly fewer cells stained positive for CD90 in this group (Fig. 5R).

### Endothelial seeding reduces ex vivo thrombogenicity of vascular grafts

**Fig. 6:**
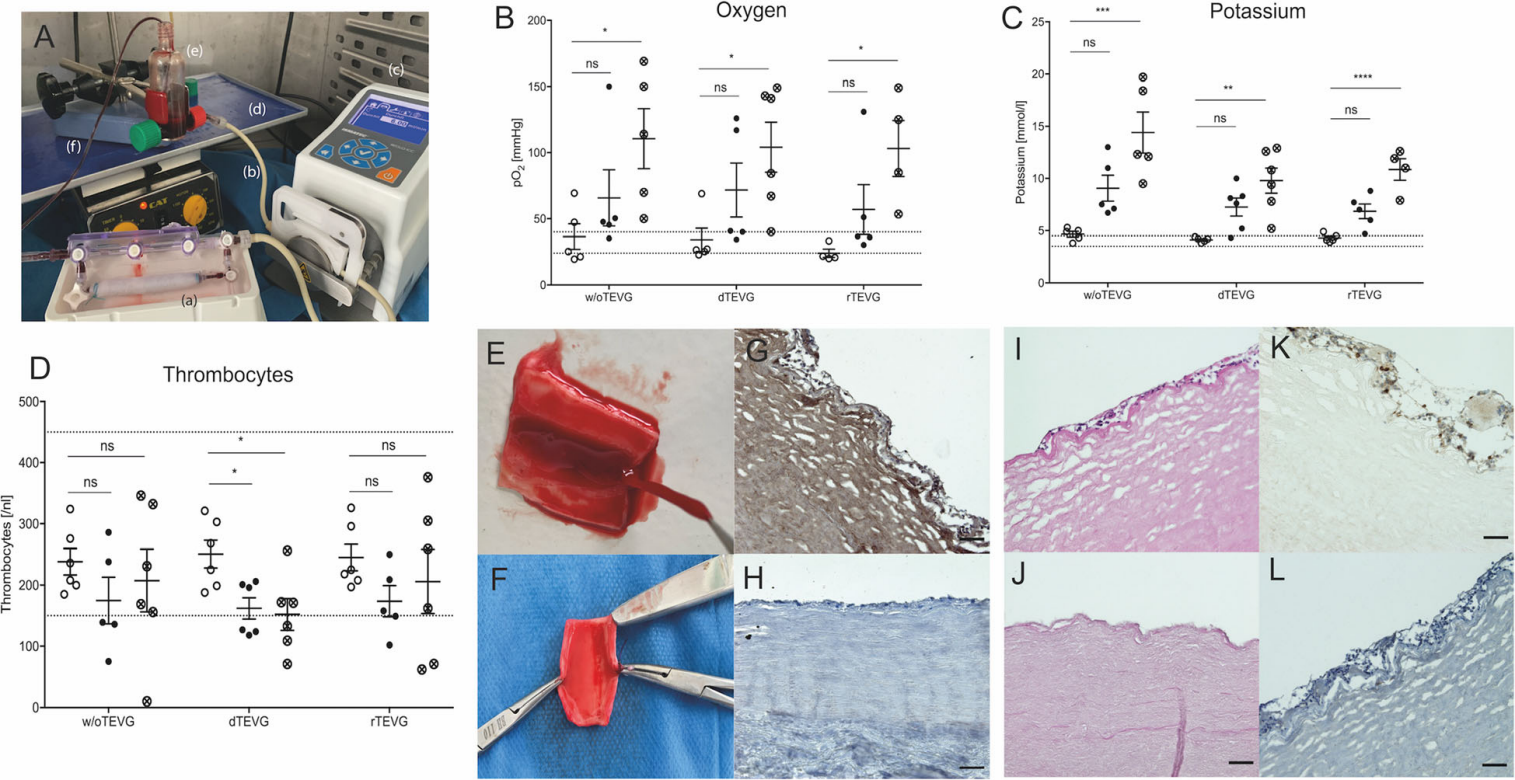
Whole blood perfusion of seeded and unseeded grafts for thrombogenicity testing. The dynamic cultivation setup was adapted for the whole blood perfusion experiments into a setup consisting of the vascular graft (a) immersed in Ringer Solution to prevent drying out, of the pump tubing (b), the peristaltic pump (c), the shaker (d), the blood-filled reservoir (e) and the plastic tubing (f) connecting the reservoir to the graft. The whole system was coated with heparin prior to starting the experiment and the shaker was set at 90 rpm throughout the perfusion (**A**). Evaluation of oxygenation prior to (t0, hollow symbol), at the first (t1, filled symbol) and second hour (t2, crossed symbol) after starting the perfusion revealed increasing oxygen levels which were significant at the second hour (**B**). Moreover, evaluation of hemolysis revealed extensively increasing K^+^ values, more enhanced in the flow circuit in absence of TEVG, but also significant in the other two groups (**C**). Evaluation of platelets as main endpoint for thrombogenicity testing indicated the seeded grafts are thromboresistant, since they induced a nonsignificant reduction in platelet while the decellularized non-seeded grafts induced a significant reduction (**D**). Macroscopic evaluation of the whole blood perfused decellularized graft revealed thrombus formation on the luminal surface (**E**), a finding that was not present in recellularized grafts (**F**). Staining of unseeded grafts with CD41 revealed platelets attached to the basal membrane, pointing to their thrombogenicity **(G)**. Seeded, whole blood perfused grafts stained negative for CD41, thus histologically confirming their thromboresistance (**H**). Since the CD41 staining of decellularized grafts revealed numerous CD41 negative cells, we repeated the H&E staining for these grafts, which revealed several acellular areas (**I**) and several cellular areas (**J**), which corresponded with the ones seen in the CD41 staining. Staining with the leukocyte common antigen CD45 revealed a large number of these cells were leukocytes, a finding that confirmed our initial hypothesis of thrombus formation (**K**). Lastly, the CD31 staining we performed to make sure these cells were not cell remnants of inadequate decellularization was, as expected, negative (**L**). Ordinary One-Way ANOVA followed by Tukey’s Multiple Comparison Test was performed (*, *p* < 0.05; **, *p* < 0.01; ***, *p* < 0.001; ****, *p* < 0.0001). Scale bar represents 50 μm

Dynamic venous flow rate whole blood perfusion experiments were performed to test thromboresistance of recellularized VG, as opposed to unseeded VG in a modified dynamic perfusion system (Fig. 6A). As already stated, a low dose heparinization of the whole blood (5 U/ml) was performed prior to perfusion. Platelet count evaluation was selected as the endpoint. To assess the extent of mechanical trauma and hemolysis, potassium and oxygen levels were evaluated at three different time points during the two-hour perfusion, both of which rose in a statistically significant manner in all three groups, as demonstrated in Fig. 6B, C. Although high pottasium levels in accordance with considerable hemolysis were expected in graft absence, (w/o TEVG; t0 vs. t2: 4.66 ± 0.60 mmol/L vs. 14.40 ± 4.41 mmol/L, *p* = 0.0007), the other two groups also showed a significant increase of pottasium levels, as displayed in Fig. 6C (dTEVG; t0 vs. t2: 4.12 ± 0.23 vs. 9.78 ± 2.96, *p* = 0.0020 mmol/L, rTEVG; t0 vs. t2: 4.3 ± 0.4 vs. 10.85 ± 2.072, *p* < 0.0001).

Most importantly, we evaluated platelet levels and extent of depletion prior to, 1 h, and 2 h after perfusion with whole recirculating blood. Circuit blockage did not occur in any of the experiments. No significant difference in platelet counts was identified after the first and second hour in absence of the graft, confirming the successful heparin coating and the feasibility of the perfusion system for further thrombogenicity studies (w/o TEVG; t0 vs. t2: 238.16 ± 52.91 n/nL vs. 207.33 ± 125.20 n/nL, *p* = 0.8363, Fig. 6D). As expected, perfusion of the dTEVG with whole blood revealed significantly reduced platelet counts at both the first and the second hour (dTEVG; t0 vs. t1: 250.66 ± 55.21 vs. 162 ± 42.93, *p* = 0.0333; t0 vs. t2: 250.66 ± 55.21 vs. 152 ± 63.79, *p* = 0.0179, Fig. 6D), while platelet depletion caused by the perfusion of the reendothelialized graft was insignificant (rTEVG; t0 vs. t2: 245.00 ± 52.85 vs. 205.33 ± 128.38, *p* = 0.7284, Fig. 6D).

Thromboresistance of recellularized grafts could also be demonstrated via representative images of the luminal surface of de- and recellularized grafts after WBP. These images reveal macroscopic thrombus formation in the lumen of unseeded grafts, but not in the lumen of seeded grafts (Fig. 6E, F). Of note, while macroscopic thrombus formation was not consistently present, microscopic thrombus was seen in all decellularized, whole blood perfused grafts. The cells attached at the luminal surface of these grafts stained positive for CD41, confirming their platelet identity. Importantly, the staining revealed lack of CD41 positive cells in recellularized TEVG, thus demonstrating their thromboresistance (Fig. 6G, H). Importantly, while staining for CD41 we noticed some CD41-negative cells that stained blue, but not brown (Fig. 6G). The H&E staining of decellularized whole blood perfused grafts also revealed acellular (Fig. 6I) and cellular (Fig. 6J) regions in the basal membrane, a finding in line with the CD41-negative cells seen in Fig. 6G. To further investigate their origin, the cells were stained with the leukocyte common antigen, which revealed multiple CD45 positive cells (Fig. 6K). These findings suggest the thrombus remained attached to the luminal surface of the decellularized grafts even after slight perfusion with Ringer solution. Moreover, staining with anti-CD31 antibody was negative, confirming these cells were not EC, and thus not remnants of an ineffective decellularization (Fig. 6L). The difference in color between the figures is related to the brightness settings in the microscope. In conclusion, while seeded grafts did not show macroscopic or microscopic thrombus formation, the unseeded decellularized grafts revealed multiple blood cells attached to the luminal surface in a discontinuous manner, some of which were identified to be thrombocytes and leukocytes via CD41 and CD45 staining respectively.

### Whole blood perfusion did not affect integrity of reconstructed endothelial monolayer

**Fig. 7:**
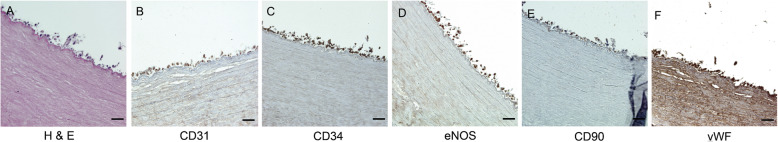
Histological and immunohistochemical evaluation of seeded vascular grafts after perfusion with whole blood. Histological evaluation of the whole blood perfused seeded grafts revealed findings similar to the seeded, non-blood perfused grafts, whereby cells were attached to the basal membrane (**A**). Staining with anti-CD31 (**B**), anti-CD34 (**C**), anti-eNOS (**D**) and anti-vWF antibody (**E**) revealed the majority of the cells were endothelial cells, while staining with anti-CD90 antibody (**F**) revealed the location of the hMSC. Scale bar represents 50 μm

Cell dislodgement is an important concern regarding ex vivo whole blood perfusion setups. Therefore, we performed histological and immunohistochemical analysis to evaluate the integrity of the endothelial monolayer and endothelial coverage of the grafts after perfusion. When assessing the stained tissue slices, we detected various grades of detachment of the monolayer from the intima, as it can be seen in Fig. 7. However, this was also the case in the recellularized grafts considered for calculation of the LECC. We hypothesized it was an artifact caused by sectioning of the paraffin blocks. Although detached from the luminal surface in some slices, histological staining with H&E revealed an intact cellular coverage of the intima (Fig. 7A). The morphology of CD31, CD34, vWF and eNOS positive hECPC and CD90 positive hMSC was similar to the non-perfused recellularized VG (Fig. 7B - F). Importantly, the mean luminal coverage of the whole blood perfused TEVG (*n* = 6) was estimated to be 61 ± 5%. Lastly, when assessing the stained tissue slices, we detected various grades of detachment of the monolayer from the intima, which was especially visible in 10 out of 43 sections. We hypothesized it was an artifact caused by sectioning of the paraffin blocks.

### Glucose consumption and lactate production as indicators of luminal coverage

**Fig. 8:**
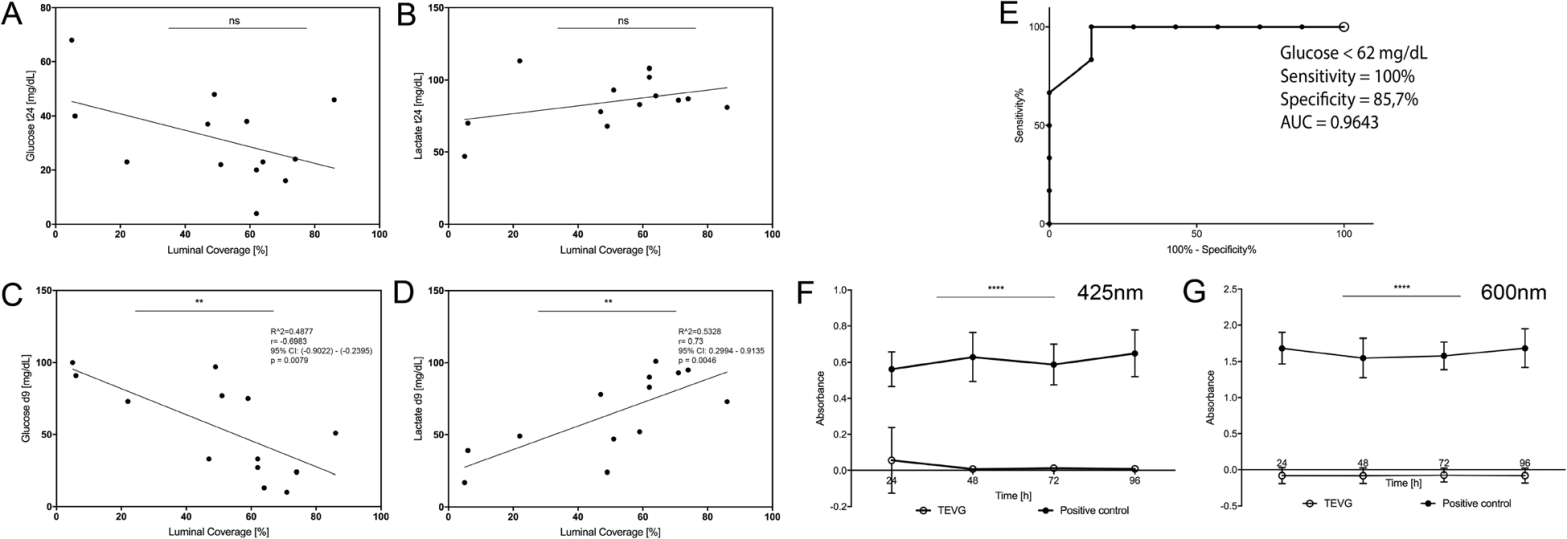
Glucose as a noninvasive indicator of luminal coverage of in vitro preconditioned TEVG. Glucose measured on the last timepoint of the static cultivation did not show a significant correlation to the estimated luminal coverage, probably due to the dynamic cultivation washing out the cells (**A**). Lactate behaved similarly (**B**). Glucose as measured on day 9 of dynamic cultivation indirectly correlated significantly to the estimated luminal coverage, confirming its validity as a noninvasive indicator (*R*^2^ = 0.4877, *r* = − 0.6983, 95% CI: − 0.9022 to − 0.2395, *p* = 0.0079) (**C**), and lactate directly correlated with the luminal coverage (*R*^2^ = 0.5328, *r* = 0.73, 95% CI: 0.2994 to 0.9135, *p* = 0.0046) (**D**). The ROC curve analysis revealed glucose greater than 62 mg/ indicates LC > 60%, with a 100% sensitivity and 87,5% specificity (Youden’s Index = 0.8571) (**E**). Lastly, bioburden analysis of the cell supernatant on the last day of cultivation revealed lack of contamination at both 425 nm (**F**) and 600 nm (**, *p* < 0.01; ****, *p* < 0.0001) (**G**)

We evaluated the LECC of 13 recellularized grafts dynamically cultivated over 10 and 14 days. As mentioned above, the estimated LECC of the 14 day perfused grafts was 33 ± 34.2%, while the mean LECC of the 10 day perfused grafts was 61.25 ± 9.10%, whereby the highest was 74% and the lowest 47%. We first investigated the potential of final glucose levels of the static culture to be used as non-invasive indicators of luminal coverage. We hypothesized they would not be an effective indicator, since subjection to long term high shear stress would ultimately cause detachment of formerly attached cells. As expected, no significant correlation was found between glucose levels of the 24 h sample (before dynamic perfusion) and luminal coverage (*R*^2^ = 0.2111, 95% CI: − 0.8063 to 0.1226, *p* = 0.1142, Fig. 8A). However, glucose levels on the day prior to finalization of the dynamic cultivation proved to be a more suited indicator. The correlation analysis revealed a statistically significant indirect correlation (*R*^2^ = 0.4877, *r* = − 0.6983, 95% CI: − 0.9022 to − 0.2395, *p* = 0.0079, Fig. 8B), showing that low glucose levels on the day before finalization of the experiment correlate with an improved LECC and can be used as a reliable non-invasive indicator of luminal coverage of the graft. The same analysis was performed for lactate production for both, static and dynamic culture. While the first correlation was statistically insignificant (*R*^2^ = 0.152, *r* = 0.3898, 95% CI: − 0.2053 to 0.7745, *p* = 0.1879, Fig. 8C), the latter revealed a statistically significant direct correlation to luminal coverage (*R*^2^ = 0.5328, *r* = 0.73, 95% CI: 0.2994 to 0.9135, *p* = 0.0046, Fig. 8D). Glucose levels lower than 62 mg/dL demonstrated the best efficacy for indicating LC > 60%, with a 100% sensitivity and 87.5% specificity (Youden’s Index = 0.8571). The area under the curve, as calculated from the ROC analysis, was 0.9643 (CI: 0.8716–1.057, *p* = 0.0053, Fig. 8E).

### Bioburden analysis

We compared the absorbance of the dynamic culture supernatant (culture medium + SOC, *n* = 13) to the absorbance of the positive control (culture medium + SOC + sputum, *n* = 3) after 24, 48, 72 and 96 h of dynamic incubation. The positive control showed constant contamination at all time points and at both 425 and 600 nm, while no contamination was detected in our experimental setup (Fig. 8F and G, *p* < 0.0001 at all time points).

## Discussion

Decisive progress regarding the availability of vascular grafts in vascular surgery in case of unavailable autologous grafts is urgently needed [6, 7]. Here we test and demonstrate the ex vivo thromboresistance of biological tissue engineered vascular grafts (TEVG) based on de- and recellularization by perfusing them with human whole blood over 2 h. **R2.2** Based on a retrospective clinical study, out of all revascularization procedures performed due to peripheral artery disease, a graft was urgently needed only in 5% of the cases [24]. Recellularizing a TEVG with autologous cells that previously need to be expanded and then dynamically conditioned in the graft for long periods of time is not appropriate for this clinical scenario. However, in most of the cases the disease develops gradually, and there are many screening methods that can be employed in low-risk and high-risk patients [25]. Thus, in many primary cases with chronic disease or cases with failure of the acutely implanted graft, there is some time available to develop the construct. We hypothesize that in these cases the isolation of the autologous cells would immediately follow at the time of diagnosis, and the surgery would be scheduled to fit the time span required for the construction of the TEVG. Finally, the implantation could be performed.

Reconstruction of the endothelial lining is decisive for long-term patency of TEVG in vivo [26–29]. However, a consensus on the most optimal way to perform cell seeding is absent [4]. *Meinhart* et al. reported that in vitro seeded ePTFE grafts perform significantly better than non-seeded synthetic grafts and show comparable patency to autologous venous grafts (64.4% vs. 68%) [30–32]. Our present study provides further insight into the topic of in vitro seeding of VG by discussing the efficacity, advantages and limitations associated with this approach. Endothelial cells are the main players in intima reconstruction [33–36]. An important 20-year-long clinical study by Herrmann et al. implemented autologous EC isolated from patient-specific vein segments to reendothelialize decellularized VG, an invasive procedure that subjected patients to additional hospitalization-associated risks such as multidrug resistant infections [37]. ECPCs have emerged as a more advantageous alternative to mature EC since they can be isolated from peripheral blood via routine venipuncture [38, 39]. Moreover, it is possible to construct an autologous VG using ECPC isolated from patients with multiple cardiovascular or other morbidities [15]. Indeed, several other important studies such as those performed **R2.4** by Hinds et al. [40, 41], Tranquillo et al. [42], and Truskey et al. [43] have implemented late-outgrowth endothelial progenitor cells in TEVG construction and subsequent biocompatibility evaluation. Herein we successfully used hECPC isolated from eight patients with various comorbidities to reconstruct the endothelial monolayer of dBCA grafts. Our immunohistochemical analysis revealed the seeded cells stained positive not only for ECPC markers, such as CD34, but also for mature EC markers, such as CD31, vWF, and eNOS (Fig. 5 A-L), corroborating with studies reporting that ECPC express mature EC markers when subjected to physiological shear stress and long-term culture [44]. Moreover, many authors report that MSC increases EC viability through the release of paracrine factors, an effect that is further enhanced under physiological shear stress [45–48]. Based on the data gathered in these studies and the data published by our group [15], we co-cultivated hEPC with hMSC derived from the umbilical cord. However, these cells are not an option when personalized TEVG are aimed. Although traditionally the bone marrow has been the most important source of MSC in humans [49], over time alternative sources such as the adipose tissue [50, 51], the salivary gland [52], and dental tissue [53] among others have also been identified [54, 55]. Especially the adipose tissue has become a very promising tissue source for allogeneic hMSC and tissue engineering, because the isolation involves only a minimally invasive procedure without major morbidities and long hospitalization. Thus, the viability of the results presented here needs to be explored with further studies where both cells are isolated from the same patient.

The in vivo performance of tissue engineered vascular grafts upon implantation depends mostly on the optimal ex vivo conditioning preceding the implantation. One of the most important aspects of optimal preconditioning is the supplementation of physiological flow and shear stress to avoid cell detachment upon encounter with blood flow and shear stress in vivo [56]. Physiological wall shear stress (WSS) values range from 10 to 16 dynes/cm^2^ for large diameter arteries (0.3 cm) and 18–26 dynes/cm^2^ for small diameter arteries (0.1–0.06 cm) [56, 57]. On the other side, the great saphenous vein registers physiological shear stress of 0.2 dynes/cm^2^ and a non-pulsatile flow [58]. In the present study we purposely subjected the vascular grafts to a subphysiological WSS of 0.033 dynes/cm^2^ during the dynamic culture, and then adjusted to physiological venous WSS of 0.6 dynes/cm^2^ during WBP (Fig. 2B). The gradually increasing shear stress achieved in the current study accounted for a histologically visible change in cell morphology and prepared the cells for the physiological shear stress they were subjected to during ex vivo WBP experiments, as demonstrated by the preserved luminal coverage. An implantation of these TEVG would subject the ECPC to abruptly changing arterial shear stress varying from less than 1 dynes/cm^2^ to 600 dynes/cm^2^ [59, 60], hypothetically leading to cell detachment. Unfortunately, it is extremely challenging to achieve more physiological arterial flow rates and shear stress in ex vivo perfusion conditions, rendering implantation experiments indispensable [61]. Indeed, our calculations show that to achieve physiological arterial WSS values while cultivating cells with cell medium, approximate flow rates ranging from 860 ml/min to 1460 ml/min are required, which translates into either abruptly increasing high flow rates with the risk of cell detachment [56] or slowly increasing flow rates over exceedingly long-term culture periods, which also leads to cell detachment (Fig. 5 M-R). Moreover, the composition of the perfusion system may also have an effect on conditioning, maturation, proliferation and functionality of the seeded cells. Indeed, the presence of a medium-containing reservoir, connecting tubes and three-way stopcocks in the perfusion system accounts for disrupted and turbulent flow. On the other side, although the peristaltic pump exerts pulsatile flow that imitates blood flow in the body, it also accounts for high mechanical trauma and extensive hemolysis. All in all, although we were able to achieve some physiological conditions for the dynamic cultivation of our TEVG, the ex vivo environment is not ideal, and the preliminary short-term conditioning that is possible within these conditions must be followed-up by an implantation.

Successful in vitro engineering of vascular grafts requires a dependable method that enables continuous noninvasive supervision of cell culture conditions and extent of cell viability, detachment or overgrowth [62, 63]. Despite some variations, our method is in line with that used by *Carrier* et al. in a study published in 1999 [64]. We implemented glucose uptake and lactate production as non-invasive indicators of cell proliferation and viability, and pH, pCO_2_, pO_2_, Na^+^, and K^+^ as indicators of cell culture stability. Regular measurement during the static seeding revealed gradually decreasing glucose levels associated with a significant increase in lactate and pCO_2_ and a significant decrease in pH (Fig. 4). While the gradual increase in pCO_2_ during static cultivation of our grafts could be explained by the Krebs cycle involved in aerobic glycolysis, the anaerobic glycolysis that occurs at insufficient oxygen levels could be the reason behind the increase in lactic acid, both generating low pH [65]. We interpreted the absence of these changes during the dynamic culture to indicate cell culture stability, lack of cell overgrowth and lack of cell detachment. The most striking result emerging from this data is the significant indirect correlation between final glucose levels of dynamic culture and the estimated percentage in luminal coverage, confirming glucose levels can be implemented as reference markers to indicate endothelial coverage of VG (Fig. 8 C, D). This lends support to previous findings in the literature, where glucose consumption rate (GCR) was used as an indicator of growth of endothelial cells and as predictor of in vivo performance [66]. In our view, the average 61% coverage estimated after selectively perfusing grafts with a predicted LECC > 60% with whole blood confirms and emphasizes the validity of our model. Importantly, the lack of correlation between static cultivation parameters and estimated LECC can be attributed to the fact that long-term dynamic culture and gradually increasing flow rates wash out a considerable amount of cells, even under low venous shear stress (Fig. 8A, B). As expected, similar findings applied to final average lactate levels, which directly correlated to percentage of LECC. Importantly, we tested the correlation between glucose and lactate levels and the LECC of the grafts on day 14; this correlation resulted insignificant (Supplementary Fig. 1C).

Graft thrombogenicity due to incomplete luminal reendothelialization is an important concern in vascular tissue engineering. Therefore it is imperative to investigate the anti-thrombogenic properties of seeded grafts ex vivo. Unfortunately, several bottlenecks stand in the way of designing an accurate experimental setup for this purpose [18]. Due to the previously mentioned logistical difficulties, many researchers have shied away from long-term high shear stress whole blood perfusion setups and focused on alternative investigation methods [36, 67–70]. However, others have tried to recapitulate whole blood flow conditions, either through shaking [71] or through exposure to dynamic blood flow using a variety of methods, such as the Baumgartner assay [72–76]. Despite the mentioned difficulties, we tried to optimally recapitulate the physiological conditions the VG would be subjected to upon implantation by perfusing preconditioned VG over 2 h with slightly heparinized human whole blood recirculating at venous shear stress in a closed-loop perfusion system. During the duration of this study two main aspects of whole blood perfusion needed to be established and were especially challenging: the duration of the perfusion and the endpoint selection. We limited the perfusion time at 2 h due to hemolysis, an inevitable event that points to mechanical trauma arising from high shear stress perfusion systems involving peristaltic pumps. K^+^ levels measured to evaluate the extent of this phenomenon in our setup revealed the hemolysis gradually increased over time, and as expected, was overall significantly more enhanced in the group without TEVG (Fig. 6C). However, pO_2_ evaluated at three time-points revealed a lack of hypoxia, which is also known to cause extensive hemolysis [77] (Fig. 6B). Prior to this, we aimed to use intravascular pressure increase due to thrombus formation as the main endpoint, but this was not possible since relevant thrombus formation required very long perfusion times up to 20 h, which was associated with extreme hemolysis.

In whole blood thrombogenicity measurements endpoint selection is crucial and should be based on the flow regime and the surfaces being compared. References from the literature suggest that high flow rate thrombogenicity studies generate the most significant differences between seeded and unseeded surfaces [18, 78]. Based on this principle, we implemented thrombocyte counts as the main endpoint to assess thromboresistance in our study. After multiple attempts, this endpoint was in agreement with the flow profile we implemented, was logistically effective and allowed us to correctly evaluate the relevance of our hypothesis. As mentioned above, we also modified the perfusion time accordingly, so that the duration did not lead to extensive cell death and hemolysis. The most conspicuous observation to emerge from the data comparison is the insignificant platelet depletion induced by the seeded vascular grafts compared to the significant depletion in unseeded TEVG, a result that has further strengthened our confidence in the vast advantages of in vitro recellularization. However, the expected significant platelet depletion both at the first and the second hour of whole blood perfusion of dTEVG is also noteworthy, since it provides further evidence for the widely acknowledged immediate thrombogenicity of exposed ECM [18]. To our knowledge, this is the first time the thromboresistance of a newly reendothelialized, biological and potentially autologous TEVG is tested in an ex vivo, long term, high flow perfusion system with human whole blood. Our values are consistent with a study by *Kaplan* et al., where a significant decrease in platelets after whole blood perfusion of uncoated PVC foils was reported, whereas perfusion of fibrinogen/heparin-coated foils did not cause a significant change [73]. Nevertheless, in contradiction with our findings, they reported insignificant changes also in other CBC parameters, such as white blood cells, hemoglobin, hematocrit, and red blood cells [73]. Although hematocrit, hemoglobin, and erythrocyte depletion were insignificant during perfusion of our seeded grafts, perfusion of unseeded grafts as well as the flow circuit itself in absence of a graft induced a significant decrease in all three parameters (Suppl. Fig. 2 A-C). Also different from the mentioned study, our results show an extensive significant depletion of white blood cells in all three groups (Suppl. Fig. 2D). The foremost cause of these discrepancies may be the considerable mechanical trauma associated with the high shear stress and use of a peristaltic pump in our study. Indeed, these factors are absent in the mentioned study, where blood flow inside the Chandler’s Loop is provided via rotation at 30 RPM, accounting for minimal mechanical stress to the cells [73].

In conclusion, this study confirms ex vivo thromboresistance of biological tissue-engineered vascular grafts preconditioned with human vascular cells. To the best of our knowledge, this is the first study to expose engineered vascular grafts to human whole blood recirculating at high flow rates. The findings indicate platelet depletion during perfusion of unseeded grafts was significantly higher when compared to the minor decrease caused by perfusion of seeded grafts. Besides confirming the stability of the reconstructed endothelial monolayer by reporting a preserved luminal coverage of more than 50% even after perfusion with whole blood, we could also demonstrate glucose as a non-invasive indicator of the graft endothelial coverage.

## Experimental limitations

The present study entails several experimental constraints, which can be classified into limitations related to in vitro preconditioning and limitations related to thrombogenicity assessment using whole blood in an ex vivo setup. Despite studies acknowledging its advantages, in vitro preconditioning remains labor-intensive, which is why this approach has lost popularity. Moreover, achieving 100% luminal coverage is a challenge and requires very high cell counts. Since studies have shown considerable recellularization also occurs post-implantation in situ and also taking as a reference a study performed by *Ott* et al. [17] we aimed at a coverage greater than 50% and achieved an average coverage of 60%. However, a long-term evaluation of response to various luminal coverage grades after implantation is required for an objective assessment. Moreover, we were unable to expose the recellularized TEVG to a physiological arterial shear stress that varies from less than 1 dynes/cm^2^ to 600 dynes/cm^2^ [59, 60], but only exposed them to sub-physiological shear stress during seeding and cultivation and venous shear stress during perfusion with whole blood. Indeed, flow rates ranging from 860 ml/min to 1460 ml/min translate into either abruptly increasing flow or slowly increasing flow over exceedingly long-term culture periods, which both lead to cell detachment. Lastly, when assessing the stained tissue slices, we detected various grades of detachment of the monolayer from the intima, which was especially visible in 10 out of 43 sections. We hypothesized it was an artifact caused by sectioning of the paraffin blocks.

The results of ex vivo whole blood perfusion experiments can be influenced by various experimental constraints [79]. The tubes constituting the flow system unavoidably provoke CBC changes unrelated to the vascular graft. Increasing the surface area of the tested graft in relation to that of connectors or coating the connecting tubes with heparin are feasible alternatives to isolate graft-specific thrombogenicity. Unfortunately, the surface area of the connectors required for our dynamic WBP setup exceeds that of our TEVG. Thus, we coated the flow circuit with heparin and evaluated thrombogenicity in absence of the graft. Importantly, we did not evaluate if the coating of the tubing further heparinized the blood. Although the impact could be irrelevant considering we implemented thrombocytes as our endpoint, this could be done by comparing clotting times before and after injection into the circuit via thromboelastography or aPTT. Secondly, inter-donor variability also impacts thrombogenicity experiments. Although platelet depletion patterns were mostly similar, the standard deviation reveals few inconsistencies, which could theoretically be resolved by repeating the experiments using blood from only one donor or pooled whole blood with matching blood group. Lastly, the bovine carotid arteries and their varying macro-architecture may have contributed to the inconsistencies in macroscopic thrombus formation on the luminal surface of the TEVG.

## Supplementary Information

**Additional file 1: Supplementary Fig. 1.** Cell culture parameters during the 14-day dynamic cultivation of seeded grafts. Daily supervision of cell culture parameters such as pH, pO_2_, pCO_2_, Na^+^ and K^+^ during the 14-day perfusion revealed mainly stable values similar to the 10-day dynamic cultivation (A). Daily supervision of glucose and lactate revealed initially decreasing glucose and increasing lactate levels, which than slowly started to return to initial levels after the 10th perfusion day (B). The correlation analysis between glucose on day 14 and estimated luminal coverage after 14-day perfusion resulted insignificant (*r* = − 0.5417, 95% CI = − 0.9635 to 0.6523, *p* = 0.2935, as did the correlation between lactate and luminal coverage (*r* = 0.7548, 95% CI = − 0.3815 to 0.9827, *p* = 0.5698. (*, *p* < 0.05) (C).

**Additional file 2: Supplementary Fig. 2.** Evaluation of complete blood count parameters after whole blood perfusion of seeded and unseeded grafts for thrombogenicity testing. Supervision of the white blood cells revealed an extensive significant depletion in all three groups (A). Although hematocrit (B), hemoglobin (C), and erythrocyte (D) depletion was insignificant during perfusion of our seeded grafts, perfusion of unseeded grafts and of the flow circuit in absence of a graft induced a significant decrease in all three parameters. (*, *p* < 0.05; **, *p* < 0.01; ***, *p* < 0.001; ****, *p* < 0.0001).

**Additional file 3: Supplementary Table 1.** Detailed characteristics of patients from whom hEPC isolation was performed. The mean age of the patients was 50 years old. 50% of the patients were diagnosed with malignancy, 25% with autoimmune disorders, and 25% with other diseases.

## Data Availability

Not applicable.
