# Supplementary figures and images for: Surface modification of decellularized bovine carotid arteries with human vascular cells significantly reduces their thrombogenicity
Source: J Biol Eng. 2021 Nov 24;15:26. doi: 10.1186/s13036-021-00277-2 (PMC8611970; doi:10.1186/s13036-021-00277-2)

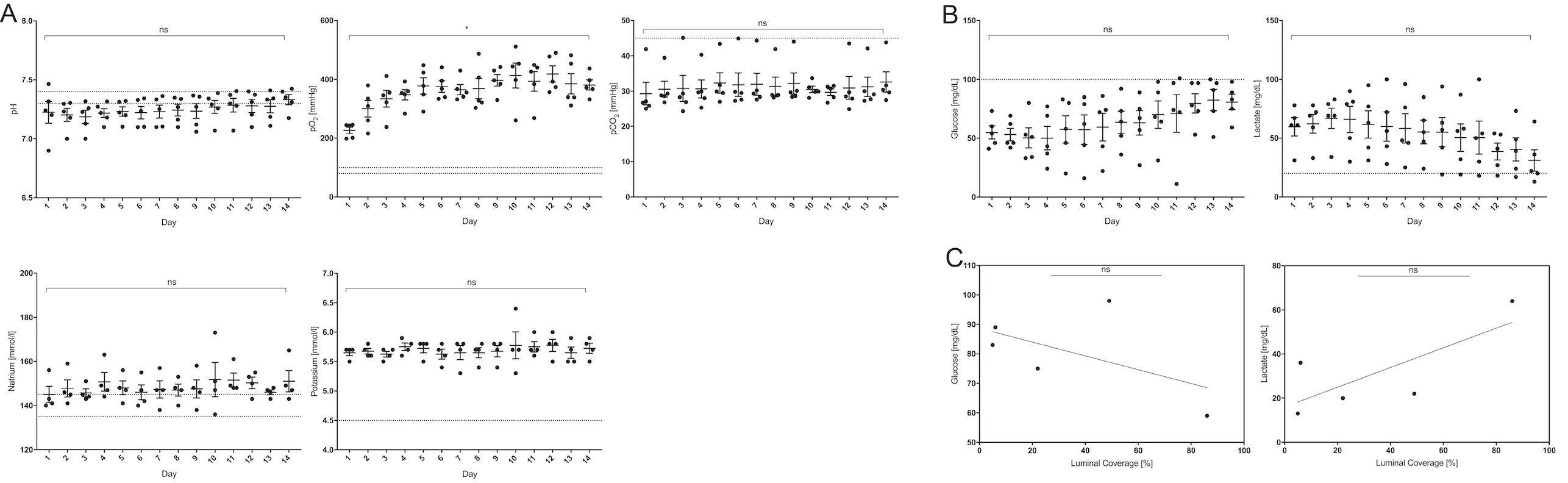

Supplement: Supplementary file 1 — Additional file 1: Supplementary Fig. 1. Cell culture parameters during the 14-day dynamic cultivation of seeded grafts. Daily supervision of cell culture parameters such as pH, pO2, pCO2, Na+ and K+ during the 14-day perfusion revealed mainly stable values similar to the 10-day dynamic cultivation (A). Daily supervision of glucose and lactate revealed initially decreasing glucose and increasing lactate levels, which than slowly started to return to initial levels after the 10th perfusion day (B). The correlation analysis between glucose on day 14 and estimated luminal coverage after 14-day perfusion resulted insignificant (r = − 0.5417, 95% CI = − 0.9635 to 0.6523, p = 0.2935, as did the correlation between lactate and luminal coverage (r = 0.7548, 95% CI = − 0.3815 to 0.9827, p = 0.5698. (*, p < 0.05) (C). [file 13036_2021_277_MOESM1_ESM.tif]

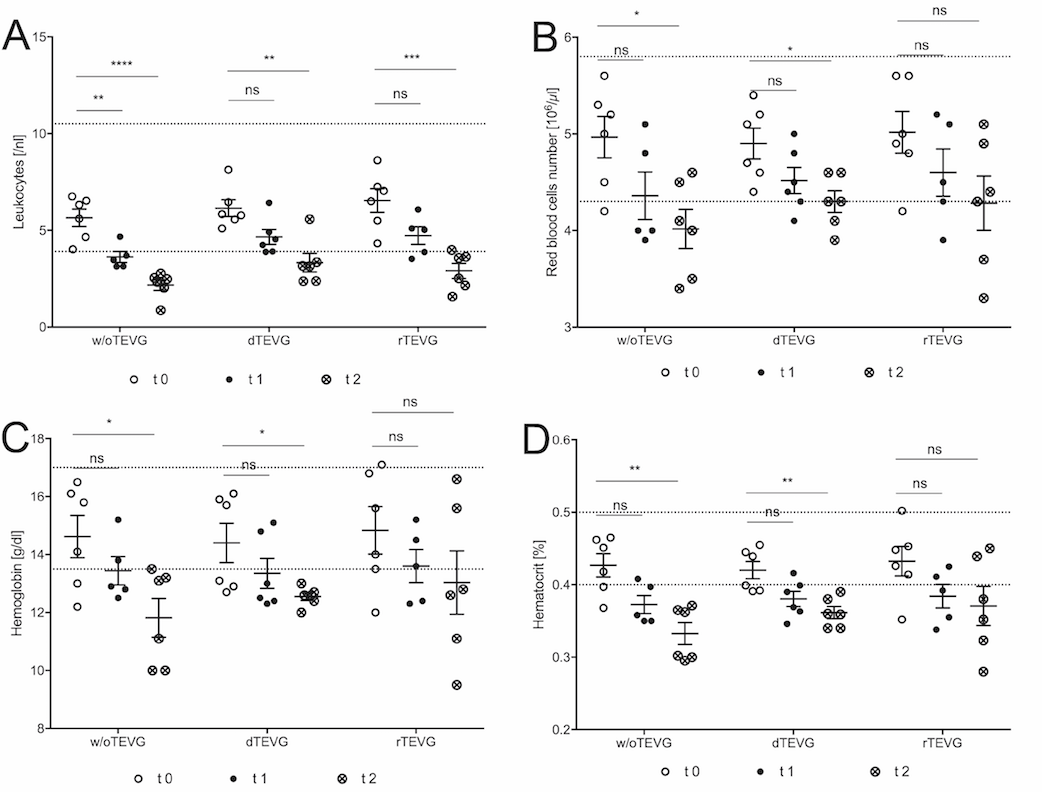

Supplement: Supplementary file 2 — Additional file 2: Supplementary Fig. 2. Evaluation of complete blood count parameters after whole blood perfusion of seeded and unseeded grafts for thrombogenicity testing. Supervision of the white blood cells revealed an extensive significant depletion in all three groups (A). Although hematocrit (B), hemoglobin (C), and erythrocyte (D) depletion was insignificant during perfusion of our seeded grafts, perfusion of unseeded grafts and of the flow circuit in absence of a graft induced a significant decrease in all three parameters. (*, p < 0.05; **, p < 0.01; ***, p < 0.001; ****, p < 0.0001). [file 13036_2021_277_MOESM2_ESM.tif]
