# Supplementary material for: Surface modification of decellularized bovine carotid arteries with human vascular cells significantly reduces their thrombogenicity
Source: J Biol Eng. 2021 Nov 24;15:26. doi: 10.1186/s13036-021-00277-2 (PMC8611970; doi:10.1186/s13036-021-00277-2)
Supplement: Supplementary file 3 — Additional file 3: Supplementary Table 1. Detailed characteristics of patients from whom hEPC isolation was performed. The mean age of the patients was 50 years old. 50% of the patients were diagnosed with malignancy, 25% with autoimmune disorders, and 25% with other diseases. [file 13036_2021_277_MOESM3_ESM.docx]

**Supplementary Table 1:** **Detailed characteristics of patients from whom hEPC isolation was performed.** The mean age of the patients was 50 years old. 50% of the patients were diagnosed with malignancy, 25% with autoimmune disorders, and 25% with other diseases.

| Characteristics (n=8) N (%) |
| --- |
| Age (median/range) 50 / 31-69 |
| Gender |
| Male 4 (50%) |
| Female 4 (50%) |
| Diagnosis |
| Malignancy 4 (50%) |
| Autoimmune 2 (25%) |
| Other 2 (25%) |
